# Serum stromal cell-derived factor-1 concentrations are increased and associated with nonalcoholic fatty liver disease in children with obesity

**DOI:** 10.1186/s12902-024-01597-2

**Published:** 2024-05-10

**Authors:** Yuesheng Liu, Lijun Hao, Linhao Wang, Mengnan Lu, Chunyan Yin, Yanfeng Xiao

**Affiliations:** 1 Department of Pediatrics, The Second Affiliated Hospital of Xi ’ an Jiaotong University, Xiwu Road, Xi ’, Shaanxi 710000 People’s Republic of China; 2 Neonatal Department, Xi’an People’s Hospital (Xi’an Fourth Hospital), Xi’an, China

**Keywords:** SDF-1, Childhood obesity, Nonalcoholic fatty liver disease

## Abstract

**Introduction:**

Stromal cell-derived factor-1 (SDF-1) is a newly discovered small molecule adipocytokine, and research has shown that it is closely related to the occurrence and development of obesity. However, there are currently few research reports on SDF-1 in childhood obesity and nonalcoholic fatty liver disease (NAFLD), and this study aims to explore the relationship between SDF-1 and obesity related indicators in obese children.

**Methods:**

Serum SDF-1 concentrations were measured using enzyme-linked immunosorbent assay (ELISA). Clinical and biochemical data were collected, such as body mass index (BMI), waist and hip circumference, blood pressure, liver enzymes, cholesterol, and fasting insulin. Children with NAFLD or not were evaluated through Color Doppler Ultrasound.

**Results:**

Serum SDF-1 concentrations were significantly higher in obese subjects than in non-obese subjects (*P* < 0.05), and were elevated in the NAFLD obese subjects than in the non-NAFLD obese subjects (*P* < 0.05). SDF-1 was positively correlated with BMI, waist-to-hip ratio, systolic blood pressure, body fat percentage (BFP), basal metabolic rate (BMR), alanine transaminase (ALT), aspartate transaminase (AST), glutyltranspeptidase (GT), and homoeostasis model of HOMA-IR, independent of their uric acid (UA), total cholesterol (TC), triglycerides (TG), high-density lipoprotein (HDL), low-density lipoprotein (LDL), very-low-density lipoprotein (VLDL), gender and age. BFP and BMR were associated with the serum SDF-1 concentrations in multivariable linear regression analysis.

**Conclusion:**

These results suggest that SDF-1 levels are elevated in obese children and are associated with NAFLD, indicating that SDF-1 may play a role in the development of childhood obesity and metabolic disorders.

## Introduction

In the past few decades, the prevalence of overweight or obesity in children has sharply increased in both developed and developing countries [1]. According to data released by the World Health Organization, as of 2016, approximately 340 million children and adolescents worldwide were overweight or obese [2]. During the recent epidemic of COVID-19, the growth rate of childhood obesity was significantly accelerated, so that a new term “Covibesity” was proposed to describe the increase in obesity rate due to the epidemic blockade [3]. Childhood obesity is associated with many complications, such as hypertension, hyperlipidemia, type 2 diabetes (T2DM), hyperuricemia and NAFLD [4].

The prevalence of NAFLD disease has increased significantly with the increase in obesity and is now the most common cause of chronic liver disease in the United States and worldwide [5]. Since Moran et al. [6] first found evidence of pediatric NAFLD in 1983, we have learned much about the characteristics of children and their potential long-term effects on health status. Fatty liver is a disease with excessive accumulation of fat in liver cells caused by various reasons. It has been reported that adult patients with fatty liver have a high possibility of developing liver fibrosis and cirrhosis [7].

As an important endocrine organ, liver secretes a series of cytokines and has a variety of receptors for cytokines. However, in morbid obesity, excessive accumulation of fat leads to hepatocyte degeneration, which disrupts the balance between cytokines and receptors and is involved in the pathophysiology and complications of fatty liver. Chemokines are small cytokines or signaling proteins secreted by cells. SDF-1 is a class of chemotactic cytokines, named CXC-chemokine ligand 12 (CXCL-12), which is a member of the CXC chemokine subfamily. CXCL-12 is continuously secreted by stromal cells, hence the designation OF CXCL-12 as stromal cell-derived factor-1. SDF-1 is widely expressed throughout the body under healthy conditions, especially through different immune cells, endothelial cells, and stem cells. In the liver environment, SDF-1 can be produced not only by biliary epithelial cells, hepatic stellate cells and hepatic sinusoidal endothelial cells, but also by malignant cells [8]. CXCR4 is the main physiological receptor for SDF-1. CXCR4 belongs to a family of seven transmembrane G-protein-coupled receptors. CXCR4 is constitutively expressed in the whole liver tissue and primary hepatocytes [9], and malignant hepatocytes can express both SDF-1 and CXCR4, suggesting that this pathway can act as an autocrine signal to stimulate cancer cell growth, migration, and invasion [10]. It was determined that CD4^+^ T cells from obese mice were more sensitive to the chemotaxis of SDF-1 [11]. According to animal experiments in mouse models, the increased affinity of SDF-1 for CXCR4 is involved in this process. CXCR4 antagonists currently under development attenuate recruitment of inflammatory cells to the inflected liver [12].

SDF-1 is a newly discovered small molecule adipocytokine, and research has shown that it is closely related to the occurrence and development of obesity. However, there are currently few research reports on SDF-1 in childhood obesity and NAFLD, and our study aims to explore the SDF-1 levels and relationship between SDF-1 and obesity related indicators in obese children and NAFLD children.

## Materials and methods

We included the study subjects based on diagnostic and exclusion criteria, divided them into groups, measured anthropometric indicators, tested serological indicators, and used statistical analysis to compare the differences between the groups.

### Study population

We enrolled a total of 107 subjects (36 girls; 71 boys) including 67 obese children and 40 nonobese children who first-time visited the Pediatric Clinic of the Second Affiliated Hospital of Xi’an Jiaotong University during December 2022 to September 2023. We defined NAFLD through liver ultrasound examination and evaluated the presence and degree of hepatic steatosis using ultrasound based on the following guidelines: (a) Diffuse hyperechoic features; (b) Deep beam attenuation; (c) Compared to the kidneys, the echo texture of the liver increases; (d) Blurred blood vessels (no normal echogenic walls in the portal and hepatic veins) [13]. The same experienced ultrasound doctor uses the Esaote MyLab 8 eXP ultrasound diagnostic system to examine the liver of obese children. We excluded children with endocrine and metabolic disorders, hereditary obesity syndrome, a history of severe heart, liver, and kidney diseases, acute or chronic infectious diseases, autoimmune diseases, malignant tumors, pituitary diseases, alcohol consumption, or taking any medication. Our study protocol was approved by the Ethical Committee of Xi’an Jiaotong University and was conducted in accordance with the Declaration of Helsinki. Written informed consent was obtained from all guardians of children participating in the study.

### General measurements

To collect the anthropometric data, children were weighed in lightweight underwear and their height is measured without shoes. Children were diagnosed as childhood obesity when their BMI (calculated as body weight/height, kg/m^2^) exceeded 95th percentile for age and sex based on data from the China Normal Weight Obesity Working Group [14]. Dividing the waist circumference (cm) by the hip circumference (cm), WHR was calculated. Blood pressures (systolic blood pressure [SBP] and diastolic blood pressure [DBP], respectively) were measured after resting for at least 10 min by automated device. BFP refers to the total body fat percentage, which was tested by bioelectrical impedance commercial glass body composition analyzer (Inbody 760, Inbody, Korea) [15]. BMR was also tested by this device [16]. Children were fasting and quiet before the measurement, then they removed their shoes and socks and wore light clothing. The participants then stood on the device while held hand grips that were slightly abducted during test.

### Biochemical measurements

Blood samples were collected after overnight fasting and centrifuged at 2000 rpm for 10 min. The serum samples were separated and stored at -80 °C until analysis. The measurements of fasting blood glucose (FPG), ALT, AST, GT, TC, TG, UA, HDL, LDL and VLDL levels were performed using an autoanalyzer (Hitachi 747; Hitachi, Tokyo, Japan). Fasting insulin was measured by chemiluminescence immunoassay (BeiFang Systems, Beijing, China). Insulin resistance was assessed by HOMA-IR, which was calculated as follows: HOMA-IR = (fasting blood glucose × fasting insulin ) / 22.5.

### Serum SDF-1 measurements

Serum SDF-1 concentrations were measured using ELISA kits (Wuhan Boster Biotechnology Inc.) according to the manufacturer’s instructions. Briefly, 100 µl of plasma samples or standards were added to each well of a 96-well plate coated with a monoclonal antibody specific for human SDF-1. After incubation for 2 h at room temperature, the wells were washed four times with wash buffer and then incubated with 200 µl of a biotinylated polyclonal antibody specific for human SDF-1 for 1 h at room temperature. After another four washes, 200 µl of streptavidin-horseradish peroxidase conjugate was added to each well and incubated for 20 min at room temperature. After a final four washes, 200 µl of substrate solution was added to each well and incubated for 20 min at room temperature in the dark. The reaction was stopped by adding 50 µl of stop solution to each well and the optical density was measured at 450 nm using a microplate reader. The serum SDF-1 concentration was calculated from a standard curve and expressed as ng/ml. The average intra- and interassay coefficients of variation (CVs) for SDF-1 were 5.3% and 6.6%, respectively.

### Statistical analysis

All statistical analyses were performed on SPSS software version 22.0 (SPSS Inc., Chicago, IL, USA). All data were assessed for normal distribution using Kolmogorov Smirnov tests. Log-transformation was employed for variables with non-normal distribution. Experiments with two groups were analyzed using t test. Correlation coefficients were calculated using the two-tailed Pearson’s correlation analysis. Multivariate linear regression analysis model were performed to evaluate the independent determinants that affected the serum SDF-1 levels. The differences were considered statistically significant at *p* < 0.05.

## Results

### The clinical characteristics

The clinical and biochemical characteristics of 40 non-obese children and 67 obese children are shown in Table 1. The average ages of non-obese children and obese children in the study were 11.15 ± 2.06 years and 11.00 ± 2.11 years, respectively. There was no significant difference in age and gender between the two groups. It can be seen that the BMI, waist circumference, WHR, SBP, DBP, TC, TG, FPG, HOMA-IR of the obese group are significantly higher than those of the control group.

**Table 1:** The clinical characteristics and biochemical features of study population

Variable	Children without Obesity (*n* = 40)	Children with Obesity (*n* = 67)
Age, years	11.15 ± 2.06	11.00 ± 2.11
Gender, female/male	22/18	49/18
Height, cm	143.12 ± 11.72	151.36 ± 9.92*
Weight, kg	33.76 ± 8.83	64.40 ± 14.66*
BMI, kg/m2	16.20 ± 2.12	27.81 ± 4.17*
Waist, cm	61.18 ± 7.51	92.87 ± 9.71*
WHR	0.81 ± 0.06	0.98 ± 0.07*
SBP, mm Hg	94.15 ± 13.73	118.47 ± 11.61*
DBP, mm Hg	57.33 ± 12.77	69.55 ± 9.41*
ALT, U/L	30.49 ± 22.71	37.77 ± 26.31
AST, U/L	27.98 ± 19.12	31.14 ± 22.98
GT, U/L	15.48 ± 3.53	23.00 ± 13.35*
UA, umol/L	302.58 ± 69.76	383.64 ± 89.62*
TC, mmol/L	3.70 ± 0.62	4.12 ± 0.72*
TG, mmol/L	1.13 ± 0.64	1.39 ± 0.66*
HDL, mmol/L	1.09 ± 0.24	1.11 ± 0.23
LDL, mmol/L	2.56 ± 0.57	2.58 ± 0.61
FPG, mmol/L	4.82 ± 0.56	5.08 ± 0.51*
Insulin, µU/ml	7.38 ± 2.52	16.89 ± 7.31*
HOMA-IR	1.59 ± 0.61	3.78 ± 1.72*
SDF-1, ng/mL	0.1301 ± 0.0599	0.1737 ± 0.0590*

Difference between two groups were analyzed by independent students t-test. Data are presented as mean ± SD.* *p* < 0.05 compared with Children without Obesity.BMI, body mass index; WHR, waist-to-hip ratio; SBP, systolic blood pressure; DBP, diastolic blood pressure; GT, glutyltranspeptidase; TC, total cholesterol; TG, triglycerides; HDL, high-density lipoprotein; LDL, low-density lipoprotein; FPG, fasting plasma glucose; HOMA-IR, homeostasis model of insulin resistance; SDF-1, stromal cell-derived factor-1

67 obese children were classified into 2 groups according to their liver color ultrasound results: obese with NAFLD group and obese without NAFLD group group. The clinical and biochemical characteristics of two groups are shown in Table 2. There was no significant difference in age, gender, BMI, WHR, SBP, DBP, TC, TG, FPG and lipoprotein between the two groups. We found that waist circumference, BMR, ALT, AST, GT, UA and HOMA-IR of the obese with NAFLD group are significantly higher than those of the obese without NAFLD group group.

**Table 2:** The clinical characteristics and biochemical features of obesity population

Variable	Children without Fatty Liver (*n* = 33)	Children with Fatty Liver (*n* = 34)
Age, years	10.93 ± 2.30	11.07 ± 1.94
Gender, female/male	25/8	24/10
Height, cm	148.44 ± 10.00	154.19 ± 9.10*
Weight, kg	60.67 ± 14.66	68.02 ± 13.93*
BMI, kg/m^2^	27.20 ± 4.48	28.40 ± 3.82
Waist, cm	88.27 ± 9.74	96.36 ± 8.23*
WHR	0.97 ± 0.08	1.00 ± 0.51
SBP, mm Hg	117.06 ± 8.64	119.79 ± 13.85
DBP, mm Hg	67.81 ± 8.82	71.18 ± 9.79
BFP, %	34.94 ± 5.44	36.53 ± 5.99
BMR, kJ/(m^2^·h)	1367.72 ± 363.30	1661.58 ± 484.48*
ALT, U/L	22.39 ± 12.21	53.15 ± 31.72*
AST, U/L	26.18 ± 6.83	36.09 ± 15.30*
GT, U/L	17.21 ± 6.59	28.62 ± 15.76*
UA, umol/L	357.58 ± 79.93	409.70 ± 98.60*
TC, mmol/L	4.00 ± 0.69	4.24 ± 0.74
TG, mmol/L	1.25 ± 0.47	1.53 ± 0.78
HDL, mmol/L	1.09 ± 0.20	1.14 ± 0.26
LDL, mmol/L	2.54 ± 0.60	2.62 ± 0.61
VLDL, mmol/L	0.38 ± 0.21	0.46 ± 0.23
FPG, mmol/L	5.02 ± 0.46	5.15 ± 0.57
Insulin, µU/ml	13.82 ± 5.15	19.86 ± 7.92*
HOMA-IR	3.11 ± 1.27	4.42 ± 1.87*
SDF-1, ng/mL	0.1535 ± 0.0452	0.1933 ± 0.0646*

Difference between two groups were analyzed by independent students t-test. Data are presented as mean ± SD.* *p* < 0.05 compared with children without obesity. BMI, body mass index; WHR, waist-to-hip ratio; SBP, systolic blood pressure; DBP, diastolic blood pressure; BFP, body fat percentage; BMR, basal metabolic rate; GT, glutyltranspeptidase; UA, uric acid; TC, total cholesterol; TG, triglycerides; HDL, high-density lipoprotein; LDL, low-density lipoprotein; VLDL, very-low-density lipoprotein; FPG, fasting plasma glucose; HOMA-IR, homeostasis model of insulin resistance; SDF-1, stromal cell-derived factor-1

### Serum SDF-1 concentrations in subgroups

**Fig. 1:**
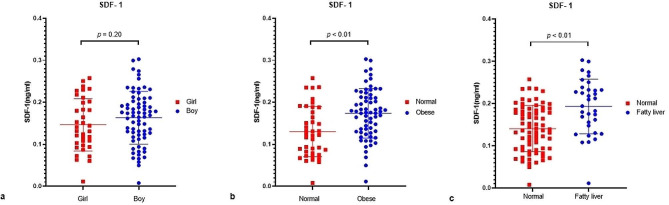
Serum SDF-1 concentrations in different gender groups (a), in obese and normal group (b), and in NAFLD and normal group (c)

We compared the levels of serum SDF-1 between different groups. There were no significant differences in serum SDF-1 concentrations between the children at different genders and ages (Fig. 1a). Serum SDF-1 concentrations were higher in obese children than in non-obese children (Table 1; Fig. 1b). Similarly, serum SDF-1 concentrations in the obese with NAFLD group were higher than that in the obese without NAFLD group group (Table 2; Fig. 1c).

### Correlations of serum SDF-1 concentrations with other variables

We investigated a potential correlation between serum SDF-1 levels and clinical variables in all subjects (Table 3). Serum SDF-1 concentrations were positively correlated with BMI (*r* = 0.240, *P* = 0.013), WHR(*r* = 0.404, *P <* 0.001), SBP (*r* = 0.343 *P* = 0.013), BFP (*r* = 0.487 *P* = 0.045), BMR (*r* = 0.508, *P <* 0.001), ALT (*r* = 0.334, *P <* 0.001), AST (*r* = 0.247, *P* = 0.010), GT (*r* = 0.270, *P* = 0.005), FPG (*r* = 0.260, *P* = 0.007), Insulin (*r* = 0.206, *P* = 0.033) and HOMA-IR (*r* = 0.213, *P* = 0.027). These results suggest that serum SDF-1 concentrations are associated with various metabolic parameters in children.

**Table 3:** Correlations of serum SDF-1 concentrations with clinical variables in all children

Variable	*r*	*p* value
**BMI**	**0.240**	**0.013**
**WHR**	**0.404**	**0.000**
**SBP**	**0.343**	**0.013**
DBP	0.180	0.064
**BFP**	**0.487**	**0.045**
**BMR**	**0.508**	**0.000**
**ALT**	**0.334**	**0.000**
**AST**	**0.247**	**0.010**
**GT**	**0.270**	**0.005**
UA	-0.055	0.659
TC	-0.076	0.436
TG	0.122	0.212
HDL	-0.157	0.107
LDL	-0.165	0.090
VLDL	0.032	0.801
**FPG**	**0.260**	**0.007**
**Insulin**	**0.206**	**0.033**
**HOMA-IR**	**0.213**	**0.027**

Pearson correlation was used for testing the correlation between continuous variables. BMI, body mass index; WHR, waist-to-hip ratio; SBP, systolic blood pressure; DBP, diastolic blood pressure; BFP, body fat percentage; BMR, basal metabolic rate; GT, glutyltranspeptidase; UA, uric acid; TC, total cholesterol; TG, triglycerides; HDL, high-density lipoprotein; LDL, low-density lipoprotein; VLDL, very-low-density lipoprotein; FPG, fasting plasma glucose; HOMA-IR, homeostasis model of insulin resistance; SDF-1, stromal cell-derived factor-1

### Multiple Linear Regression Analysis of Factors Associated with serum SDF-1 concentrations

The results of multiple linear regression analysis of factors associated with SDF-1 in all subjects are shown in Table 4. The variables that showed significant correlation with serum SDF-1 concentrations in the univariate analysis were entered into the regression model, including BMI, WHR, SBP, BFP, BMR, ALT, AST, GT, FPG, HOMA-IR. The regression model was adjusted for age and sex as covariates. We identified that BFP (β = 0.004, *P* = 0.038) and BMR (β = 0.000, *P* = 0.012) were independently associated with serum SDF-1 concentrations.

**Table 4:** Multiple linear regression analysis of clinical variables affecting serum SDF-1 levels in all children

Variable	B	SE	Standard B	t	*p* value
Constant	0.142	0.269		0.530	0.600
BMI,	0.000	0.005	0.011	0.041	0.967
WHR	0.044	0.161	0.050	0.271	0.789
SBP	-0.001	0.001	-0.177	-1.035	0.309
**BFP**	**0.004**	**0.002**	**0.314**	**2.148**	**0.038**
**BMR**	**0.000**	**0.000**	**0.389**	**2.658**	**0.012**
ALT	0.000	0.001	-0.008	-0.018	0.986
AST	0.001	0.002	0.168	0.393	0.697
GT	0.000	0.001	0.035	0.141	0.889
FPG	0.021	0.020	0.164	1.035	0.309
HOMA-IR	-0.002	0.007	-0.045	-0.266	0.792

BMI, body mass index; WHR, waist-to-hip ratio; SBP, systolic blood pressure; BFP, body fat percentage; BMR, basal metabolic rate; GT, glutyltranspeptidase; FPG, fasting plasma glucose; HOMA-IR, homeostasis model of insulin resistance

## Discussion

Worldwide, the prevalence of overweight and obesity in children has dramatically increased [2]. One of the most common complications and comorbidities of obesity is NAFLD, which has become the most common cause of childhood chronic liver disease in developed countries [17]. At present, the overall effect of prevention and treatment of childhood nonalcoholic fatty liver is still not ideal. The underlying cause is that the molecular mechanism of its development is still not fully understood.

Obesity is considered to be a low-grade chronic inflammatory state that involves a network of chemokines that contribute to a variety of diseases including NAFLD. Chemokines are a group of structurally related small molecules (8–14 kDa) that regulate the transport of various types of leukocytes by interacting with a group of 7 transmembrane G-protein-coupled receptors [18]. SDF-1 is a member of the chemokines family, expressed in various tissues and organs throughout the body, and involved in various biological and pathophysiological processes, such as embryonic development, immune escape, inflammatory response, angiogenesis, tumor occurrence, tumor migration, and tumor invasion [11, 18, 19]. There is little data from patients on the specific role of SDF-1 in obesity. However, in mouse models, J.I. Fenton [20] found that the most of the chemokines decreased in the obese group, including that serum SDF-1 concentration was increased in lean mice compared to the control mice. In our study, we found that the serum SDF-1 were increased in Chinese obese children. At present, there are almost no research reports of SDF-1 in obese children, and our study is currently the first time report of SDF-1 in Chinese children.

Our study also found that SDF-1 increased in obese children with NAFLD than those without NAFLD. There is little data from patients regarding the specific role of SDF-1 in acute liver disease. However, SDF-1/CXCR4 has been studied in mouse models, suggesting that SDF-1 plays an important role in regulating regeneration after acute liver injury [21]. It is well known that excessive obesity can cause NAFLD. Current studies suggest that SDF-1 plays an important role in the pathogenesis of NAFLD. These findings suggest that SDF-1 is involved in the occurrence of fatty liver and may be a new NAFLD biomarker. We consider that further studies are needed to investigate whether fatty liver status affects circulating SDF-1 levels in children.

We also observed that circulating SDF-1 was positively correlated with BMI, WHR, blood pressure, BFP, BMR, ALT, AST, GT, FPG, HOMA-IR, and was not correlated with age, uric acid, total cholesterol, and lipoprotein. Christian Jung [22] demonstrated that 48% of male Caucasian adolescents aged 13–17 years had a waist circumference above 90th age percentile and SDF-1 correlated inversely with the waist circumference by Linear regression analysis. Their result appears to be contrary to our study findings because we observed the results from obese and normal Chinese children, while Christian Jung observed from Caucasian adolescents. Race, age, diet, degree of objectivity and complications may all affect the results. But it all indicates that SDF-1 as a potential factor may be involved in the occurrence of obesity related complications, which worth further research. Dayea Kim’s study [23] showed that the expression and secretion of SDF-1 increased in adipose tissue of obese mice, and blocking the effect of SDF-1 reduced the aggregation of macrophages in adipose tissue, ultimately improving the systemic insulin sensitivity of insulin-resistant mice. Jihoon Shin [24] demonstrated that SDF-1 is an insulin desensitization factor in adipose cells and is overexpressed in fasting and obese adipose tissues. Exogenous SDF-1 can induce ERK signaling, phosphorylation and degradation of insulin receptor substrate-1 (IRS-1) protein in adipocytes, and reduce insulin mediated signaling and glucose uptake. On the contrary, the downregulation of endogenous SDF-1 or inhibition of its receptors in adipocytes significantly increases the level of IRS-1 protein and enhances insulin sensitivity. Dietary intake patterns are a key factor affecting obesity and complications, such as De Novo lipogenesis influenced by high fructose and high sugar intake, are also associated with NAFLD and insulin resistance. Although our study found that SDF-1 was positively correlated with HOMA-IR. its mechanism of action still needs further research.

Our study also found that BFP and BMR may affect the circulating SDF-1 levels by multivariate linear regression analysis. Indeed, obesity contributes to chronic inflammation, leading to the development of NAFLD [17]. BFP can reflect the proportion of fat in the body, and an increase in body fat percentage is inevitably related to obesity. Excessive body fat percentage can also lead to liver steatosis, leading to the occurrence of fatty liver disease. A previous study showed that CXCR4 is the primary physiological receptor for SDF-1, expressed on both adipocytes and liver cells [9]. Previous studies have shown that obese and NAFLD children are in a hypermetabolic state, characterized by an increase in basal metabolic rate, which is consistent with our study [25]. Additionally, we found that BMR is also an independent influencing factor for SDF-1. To some extent, BFP is related to adipose tissue distribution, while BMR is related to lipid metabolism. The distribution and metabolism of adipose tissue are influenced by various factors. We speculate that SDF-1 may affect the adipose tissue distribution and lipid metabolism through certain pathways, such as liver fat metabolism.

Our research still has some limitations. Firstly, our study was conducted in a single medical center, which may introduce selection bias and confounding factors. Secondly, the sample size is relatively small, which may limit the generalizability and statistical persuasiveness of the results. Thirdly, there are still some other confounding factors that may affect plasma SDF-1 levels or NAFLD, such as race, genetic heterogeneity, puberty, dietary patterns or physical activity. Fourthly, our study is only a cross-sectional survey and cannot yet elucidate the causal relationship between plasma SDF-1 levels and NAFLD. In conclusion, our study found for the first time that serum SDF-1 levels are elevated in obese children, especially in obese children with fatty liver disease. We have reason to assume that SDF-1 may play a role in the childhood obesity and metabolic disorders, which need further research to confirm its molecular mechanism.

## Data Availability

The data that support the findings of this study are available from the corresponding author upon reasonable request. The data are not publicly available due to privacy or ethical restrictions.
